# LPA suppresses HLA-DR expression in human melanoma cells: a potential immune escape mechanism involving LPAR1 and DR6-mediated release of IL-10

**DOI:** 10.1038/s41401-024-01373-x

**Published:** 2024-08-26

**Authors:** Enikő Major, Kuan-Hung Lin, Sue Chin Lee, Krisztina Káldi, Balázs Győrffy, Gábor J. Tigyi, Zoltán Benyó

**Affiliations:** 1 Institute of Translational Medicine, Semmelweis University, Budapest, Hungary; 2 HUN-REN-SU Cerebrovascular and Neurocognitive Disease Research Group, Budapest, Hungary; 3 Department of Physiology, University of Tennessee Health Science Centre, Memphis, TN USA; 4 Institute of Plant and Microbial Biology, Academia Sinica, Taipei, Taiwan China; 5 Department of Physiology, Semmelweis University, Budapest, Hungary; 6 Department of Bioinformatics, Semmelweis University, Budapest, Hungary; 7 Department of Biophysics, Medical School, University of Pecs, Pecs, Hungary; 8 Institute of Molecular Life Sciences, HUN-REN Research Centre for Natural Sciences, Budapest, Hungary

**Keywords:** lysophosphatidic acid (LPA), melanoma, death receptor 6 (DR6), IL-10, LPAR1, HLA-DR

## Abstract

While immune checkpoint inhibitors (ICIs) are promising in the treatment of metastatic melanoma, about half of patients do not respond well to them. Low levels of human leukocyte antigen-DR (HLA-DR) in tumors have been shown to negatively influence prognosis and response to ICIs. Lysophosphatidic acid (LPA) is produced in large amounts by melanoma and is abundantly present in the tumor microenvironment. LPA induces the release of various cytokines and chemokines from tumor cells, which affect cancer development, metastasis, and tumor immunity. In the present study, we investigated the role of LPA-induced IL-10 release in regulating HLA-DR expression and the underlying mechanisms in human melanoma cells. We showed that LPA (0.001–10 μM) dose-dependently increased DR6 transcript levels through activating LPAR1 in HEK293T cells. Knockdown of NF-κB1 abrogated the LPA-increased DR6 expression without affecting basal DR6 expression in both A2058 and A375 melanoma cell lines. LPA (10 µM) significantly increased IL-10 transcripts in A2058 and A375 melanoma cells, the effect was abolished by pharmacological inhibition of LPAR1 or knockdown of DR6. We found a statistically significant correlation between the expression of LPAR1, DR6 and IL-10 in human melanoma tissue and an association between increased expression of LPAR1 and reduced effectiveness of ICI therapy. We demonstrated that LPA (10 µM) markedly suppressed HLA-DR expression in both A375 and A2058 melanoma cells via activating the LPAR1-DR6-IL-10 pathway. These data suggest that the LPAR1-DR6-IL-10 autocrine loop could constitute a novel mechanism used by tumor cells to evade immunosurveillance by decreasing HLA-DR expression.

## Introduction

In the western world, the incidence of melanoma, which severely impacts patients’ life expectancy and quality of life, is continuously increasing [1]. A better understanding of the molecular mechanisms of tumor progression and anti-tumor immune response is essential to improve existing therapies and develop new ones for patients with melanoma and other cancers. Lysophosphatidic acid (LPA) is a bioactive lipid mediator produced in large amounts by melanoma that is abundantly present in the tumor microenvironment. LPA regulates a wide range of physiological and pathological cellular functions in almost every cell type via six cognate G-protein coupled receptors (LPAR1-6), which can activate several intracellular signaling pathways [2]. In the plasma, LPA is generated primarily from circulating pools of lysophosphatidylcholine and other lysophospholipids by the lysophospholipase D activity of the enzyme autotaxin (ATX), encoded by the human *ENPP2* gene [3]. Interestingly, ATX was first isolated from human A2058 melanoma cells as an “autocrine motility factor” [4]. The expression of ATX and LPAR is upregulated in several cancer types, where they mediate various aspects of carcinogenesis, including proliferation, survival, migration, angiogenesis, metastasis, and inflammation [5]. In clinical studies, accelerated tumor progression and metastasis formation are associated with high levels of ATX, which results in elevated levels of LPA [6]. Furthermore, LPA induces the release of various cytokines and chemokines from tumor cells, which can affect cancer development, metastasis, and tumor immunity [7, 8].

Death receptor 6 (DR6, encoded by the human *TNFRSF21* gene) is a recently identified type I transmembrane receptor that belongs to the tumor necrosis factor superfamily. DR6 mRNA is expressed in various organs, including the brain, heart, pancreas, and placenta [9]. Since elevated expression of DR6 plays a pivotal role in numerous human diseases, including Alzheimer’s disease (AD), inflammation, and autoimmune disease, it is considered to be a potential therapeutic target [9]. Similarly, abundant transcript levels of DR6 were observed in several human cancers, suggesting a potential role in tumor biology [10–12]. For example, upregulation of DR6 expression in lung cancer promotes tumor aggressiveness [13], while low expression of DR6 increases the overall survival probability in pancreatic adenocarcinoma [14]. Furthermore, Yang et al. showed that DR6 is required for tumor angiogenesis in B16 murine melanoma through the induction of IL-6 via NF-κB-dependent signaling [15]. DR6 has also been implicated in pro-apoptotic signaling [16]. Interestingly, Dong et al. demonstrated that LPA is able to induce apoptosis in HeLa cells via the upregulation of DR6 [16], although other investigators did not confirm this effect under the same conditions [17].

Interleukin 10 (IL-10) is primarily recognized as an anti-inflammatory cytokine secreted by immune cells, although it has been subsequently discovered that IL-10 can also be produced by non-immune cell types, including fibroblasts and keratinocytes, and various tumors, such as breast, colon carcinoma, and melanoma [18]. Although the role of IL-10 in tumor biology is controversial, elevated serum levels of IL-10 are reportedly associated with a poor prognosis in melanoma [19–21]. Furthermore, abundant IL-10 expression is accompanied by an increase in other inflammatory mediators and its expression worsens the outcomes of various cancers, suggesting that IL-10 can serve as a key regulator of tumor immunity [22, 23].

Tumors can evade immunosurveillance through several mechanisms, including altering the expression of immunologically relevant cell surface molecules and/or creating an immunosuppressive microenvironment through the accumulation of metabolites, cytokines, and chemokines. Although immune checkpoint inhibitors (ICIs) have improved survival rates for melanoma, approximately half of patients with metastatic melanoma do not respond well to ICIs [24]. Strikingly, melanomas with low expression levels of the human leukocyte antigen DR (HLA-DR) respond poorly to immunotherapies [25].

Investigating the relationship between LPA-LPAR signaling and IL-10-HLA-DR expression represents a topic previously unexplored in melanoma research. As elevated IL-10 levels are frequently associated with poor prognosis in different cancer types, we aimed to examine the potential role of LPA-induced IL-10 release in regulating HLA-DR expression and clarify the underlying signaling mechanisms in human melanoma cells.

## Materials and methods

### Reagents

LPA 18:1 was purchased from Avanti Polar Lipids Inc (Alabaster, AL, USA) and dissolved in 1 mM fatty acid-free bovine serum albumin (BSA; Merck KGaA; Darmstadt, Germany). AM095 [26], Ki16425 [27], and pertussis toxin were obtained from Cayman Chemicals (Ann Arbor, MI, USA).

### Cell culture

Human embryonic kidney HEK293T (RRID: CVCL_0063) cells, human melanoma A2058 (RRID: CVCL_1059), and A375 (RRID: CVCL_0132) cells were purchased from the American Type Culture Collection (Rockville, MD, USA). The cell lines were maintained in Dulbecco’s modified Eagle’s medium (DMEM) supplemented with 10% fetal bovine serum (FBS) and 1% Penicillin/Streptomycin and were cultured in a humidified incubator at 37 °C and 5% CO_2_. Each cell line underwent regular mycoplasma screening and all experiments were performed using mycoplasma-free cells.

### LPA treatment

In all experiments, cells were serum-starved for 1 h prior to the administration of LPA. For inhibition of LPAR, the cells were pretreated with AM095 or Ki16425 at 10 µM for 30 min prior to LPA treatment. To investigate G_i_ protein coupling, the cells were preincubated with 100 ng/mL pertussis toxin (PTX) for 16 h prior to LPA treatment. Anti-IL-10 neutralizing antibody (JES3-9D7) or IgG1 isotype control (Thermo Fisher Scientific) was administered at 3.5 µg/mL, 1 h prior to LPA treatment.

### Luciferase assay

Genomic DNA was isolated from human keratinocytes using DNeasy Blood & Tissue kit (Qiagen) and used as a template to amplify the predicted hDR6 promoter using forward and reverse primers with the sequences 5′-TCCATCGAGCTCTTGGGGGAAGGGTGATTAAA-3′ and 5′-AAAACTCGAGTTCTGCCCAGCGCCGCATCCACC-3′, respectively. The amplicon was cloned between the *Sac* I and *Xho* I restriction sites of the pGL4.10 luciferase expression vector (Promega, Madison, WI, USA). All constructs were confirmed by DNA sequencing.

HEK293T cells were cultured in 96-well plates. Twenty-four hours after seeding, the cells were co-transfected with the hDR6p-pGL4.10-luc firefly luciferase expression vector and a pRL *Renilla* luciferase control reporter driven by a simian virus 40 (SV40) promoter (Promega). Plasmid transfection was performed using Lipofectamine3000 (Invitrogen, Karlsruhe, Germany) in OptiMEM medium (Invitrogen) without supplements, according to the manufacturer’s protocol. After 24 h, cells were kept in a serum-free medium for 1 h and treated with 10 µM LPA or its vehicle for the indicated times. Luciferase activities were measured using the Dual-Glo Luciferase Reporter Assay System (Promega) according to the manufacturer’s instructions. The relative activity of firefly luciferase was calculated by normalizing it to the activity of *Renilla* luciferase.

### Gene knockdown

Small interfering RNA (siRNA) targeting DR6 (Catalog ID: L-004450-00-0005), IL-10 (Catalog ID: L-005066-00-0005) or NF-κB1 (Catalog ID: L-003520-00-0005) mRNA (ON-TARGET*plus* SMARTpool) and non-targeting control siRNAs (siNC) were purchased from Dharmacon (Lafayette, CO, USA). siRNAs were applied at the time of cell plating at a 25 nM final concentration using Lipofectamine RNAiMAX (Invitrogen), according to the manufacturer’s instructions. Treatments and measurements were performed 24 h after transfection.

### Quantitative RT-PCR (RT-qPCR)

RNA was isolated from cells using the NucleoSpin RNA Plus XS kit (Macherey-Nagel GmbH & Co. KG; Düren, Germany). RNA concentration and quality were assessed using a Nanodrop spectrophotometer (Thermo Fisher Scientific). Up to 1 µg of total RNA was converted to cDNA using a RevertAid First Strand cDNA Synthesis kit (Thermo Scientific). RNA expression relative to that of GAPDH was assessed by quantitative PCR (qPCR) using cDNA corresponding to 40 ng input RNA. Reactions were performed with 250 nM of each forward and reverse primers in a final volume of 10 µL of 2× SsoAdvanced Universal SYBR Green Supermix (BioRad, Hercules, CA, USA). After one initial denaturation step (3 min at 98 °C), amplification was performed for 40 cycles at 95 °C/10 s and 60 °C/20 s using a CFX Connect™ Real-Time PCR Detection System (BioRad). The fold change of DR6 or IL-10 mRNA expression normalized to the housekeeping gene (GAPDH) in LPA-treated versus untreated control cells was defined as 2^-ΔΔCT^. The primer sets used were as follows: GAPDH Fwd: 5′-TCGGAGTCAACGGATTTG-3′, Rev: 5′-CAACAATATCCACTTTACCAGAG-3′; DR6 Fwd: 5′-GGCATGAACTCAACAGAATC-3′, Rev: 5′-GTTGACTACCTGAAGGTTTG-3′; IL-10 Fwd: 5′-GCCTTTAATAAGCTCCAAGAG-3′, Rev: 5′-ATCTTCATTGTCATGTAGGC-3′ (Merck KGaA; Darmstadt, Germany).

### ELISA

Supernatants from melanoma cell cultures were collected after 12 h of 10 µM LPA treatment and their IL-10 content was quantified using the Human IL-10 ELISA kit (Abcam), according to the manufacturer’s instructions.

### Flow cytometry

Cells were washed and resuspended in PBS supplemented with 1% BSA and stained with antibodies specific for DR6 (7678R, Bioss) or HLA-DR (LN3, Invitrogen) at 4 °C for 30 min. At least 2 × 10^4^ events per sample were counted using flow cytometry (CytoFLEX, Beckman Coulter Life Sciences; Indianapolis, IN, USA). The resulting data were analyzed using CytExpertCell software (Beckman Coulter Life Science).

### Analysis of gene expression in melanoma samples

A transcriptome database of immunotherapy-treated patient samples had been established previously [28]. The gene expression data were quantile normalized to integrate datasets generated using different technologies. From the entire database, only samples from melanoma patients treated with anti-PD-1 therapies, specifically nivolumab or pembrolizumab, were included. To increase the sample size and robustness of the analysis, we included deidentified data from all available patients, irrespective of tumor histology. However, to avoid the confounding effects of ongoing systemic immune modulation, only pre-treatment samples - those obtained before the initiation of immune therapy - were used to evaluate the correlation between LPAR1 expression and the effectiveness of anti-PD-1 therapy.

### Statistical analysis

All data were obtained from at least three independent experiments and are presented as mean values ± the standard error of the mean. Statistical analysis was performed with Prism 6 software (GraphPad Software Inc.; La Jolla, CA, USA), using one-way ANOVA and Dunnett’s post hoc test. Statistical significance was considered at *P* < 0.05. The correlation analysis was performed by computing the Spearman rank correlation. To evaluate the correlation with therapy response, receiver operating characteristic analysis was performed and the area under the curve (AUC) value was calculated to determine the overall predictive effect.

## Results

### Effect of LPA on DR6 receptor expression

**Fig. 1:**
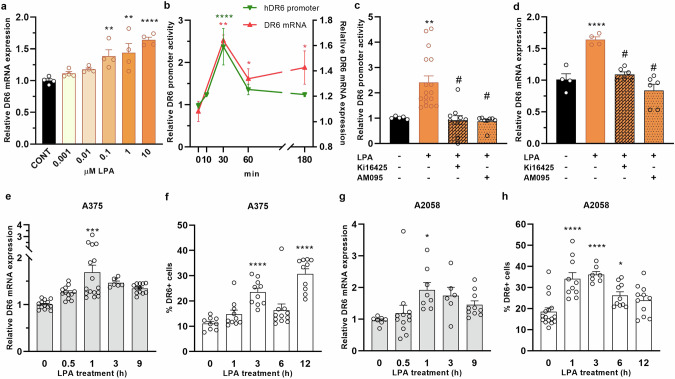
LPA-induced upregulation of DR6. **a** HEK293T cells were treated with the indicated concentrations of LPA for 30 min and DR6 expression was measured using qPCR. **b** HEK293T cells transfected with the DR6 promoter construct were treated with 10 µM LPA for the indicated times and luciferase activity was measured (green line). Relative expression of the DR6 transcript was analyzed by qPCR (red line). **c** HEK293T cells transfected with DR6 promoter construct were treated with 10 µM LPA for 30 min. Where indicated, cells were pretreated with either LPAR1/3 inhibitor Ki16425 (10 µM) or LPAR1 inhibitor AM095 (10 µM). **d** The inhibition of the LPA-induced upregulation of DR6 by Ki16425 or AM095 was confirmed by qPCR. **e**–**h** Melanoma cells were treated with 10 µM LPA. Time-dependent induction of DR6 expression was followed by qPCR (**e**, **g**) and flow cytometry (**f**, **h**). Statistical analysis was performed using one-way ANOVA and Dunnett’s posthoc test; *n* = 3–17, *P < 0.05, **P < 0.01, ***P < 0.001, ****P < 0.0001 vs. control, ^#^*P* < 0.0001 vs LPA alone.

First, we examined the effect of LPA on the expression of the DR6 receptor in HEK293T cells and found that LPA treatment resulted in upregulation of DR6 transcript levels in a dose-dependent manner (Fig. 1a). To investigate the effect of LPA on DR6 promoter activity, we constructed a plasmid in which expression of luciferase was directed by the human DR6 promoter, used it to transfect HEK293T cells, and after incubation measured luciferase activity (Supplementary Fig. S1). We found that a 30-min LPA treatment increased the DR6 promoter activity relative to that of the vehicle-treated control and that the expression of endogenous DR6 mRNA also increased with a similar time course (Fig. 1b). Interestingly, while promoter activity declined after 60 min, the DR6 mRNA levels remained elevated even 3 h after LPA stimulation.

We next sought to identify the receptor that was mediating this effect of LPA. When expression of the six LPA GPCRs was examined in HEK293T cells, LPAR1 and LPAR3 exhibited the highest expression levels (Supplementary Fig. S2). Thus, we tested whether inhibitors specific for either LPAR1/3 (Ki16425) or LPAR1 (AM095) can interfere with this effect of LPA. Both Ki16425 and AM095 inhibited the effect of LPA on DR6 promoter activity (Fig. 1c) and mRNA levels (Fig. 1d), suggesting that LPAR1 mediates the LPA-dependent upregulation of DR6 expression.

To explore the potential role of the LPA-DR6 axis in melanoma, we tested the ability of LPA to induce the expression of DR6 in human A375 (Fig. 1e, f) and A2058 melanoma cells (Fig. 1g, h). We found that LPA treatment increased DR6 mRNA and protein levels in both melanoma cell lines. LPA-induced DR6 mRNA transcript levels reached a maximum level 1 h after LPA treatment (Fig. 1e, g), whereas the expression of the DR6 receptor on the cell surface peaked at 3 h after LPA treatment in both melanoma cell lines (Fig. 1f, h). Interestingly, in the A375 cell line, LPA induced biphasic expression of the DR6 protein, with a second increase occurring 12 h after LPA treatment (Fig. 1f).

### Signaling of LPA-induced DR6 upregulation in human melanoma cell lines

**Fig. 2:**
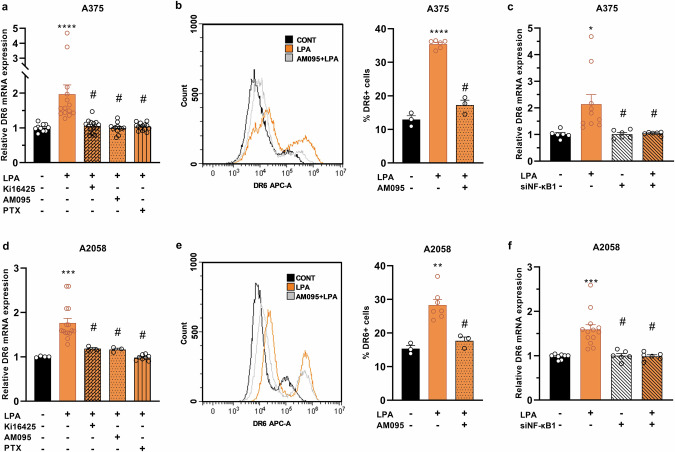
Activation of the G_i_-coupled receptor LPAR1 increases DR6 expression of melanoma cells in an NF-κB-dependent manner. Human melanoma cells were treated with 10 µM LPA for 1 h in the presence of 10 µM Ki16425 or 10 µM AM095. The pretreatment with PTX (100 ng/mL) was given 16 h prior to the LPA treatment. Gene expression was evaluated by qPCR (**a**, **d**). The inhibitory effect of AM095 on DR6 protein expression was analyzed by flow cytometry (**b**, **e**). NF-κB1 was silenced using specific siRNA to assess its role in the induction of DR6 by LPA (**c**, **f**). Statistical analysis was performed using one-way ANOVA and Dunnett’s posthoc test; *n* = 3–13, *P < 0.05, **P < 0.01, ***P < 0.001, ****P < 0.0001 vs. control; ^#^*P* < 0.001 vs. LPA alone.

First, we analyzed the relative expression levels of various LPA receptors in human A375 and A2058 melanoma cells as representatives of primary and metastatic melanoma models, respectively, and found that both cell lines expressed predominantly LPAR1 and LPAR3 (Supplementary Fig. S3). Inhibiting the LPAR1/3 receptors with Ki16425 or selectively inhibiting LPAR1 with AM095 completely abolished LPA-induced DR6 mRNA expression in both cell lines, supporting a central role of LPAR1 in this process (Fig. 2a, d). To further validate these findings, we used flow cytometry analysis to show that LPA induced a marked increase in the level of DR6 protein, which was inhibited by AM095 in both A375 (Fig. 2b) and A2058 cells (Fig. 2e).

To examine the G-protein coupling of LPAR1 to DR6 upregulation, melanoma cells were pretreated with pertussis toxin (PTX), a specific inhibitor of G_i_. PTX treatment abrogated the effect of LPA in both melanoma cell lines (Fig. 2a, d). Using ALGGEN PROMO software for prediction of transcription factor binding sites we found that the putative promoter sequence of DR6 contains binding sites for the transcription factor NF-κB1 **(**Supplementary Fig. S1). Since LPA is a known activator of NF-κB, we investigated its involvement in LPA-induced DR6 expression. We found that siRNA silencing of NF-κB1 abrogated the LPA-induced DR6 expression without affecting basal DR6 expression (i.e., in the absence of LPA) (Fig. 2c, f). These results indicate that stimulation of the G_i_-coupled-LPAR1 by LPA increases DR6 expression via activation of NF-κB1 in both A2058 and A375 melanoma cell lines.

### Effect of LPA on IL-10 production in human melanoma cell lines

**Fig. 3:**
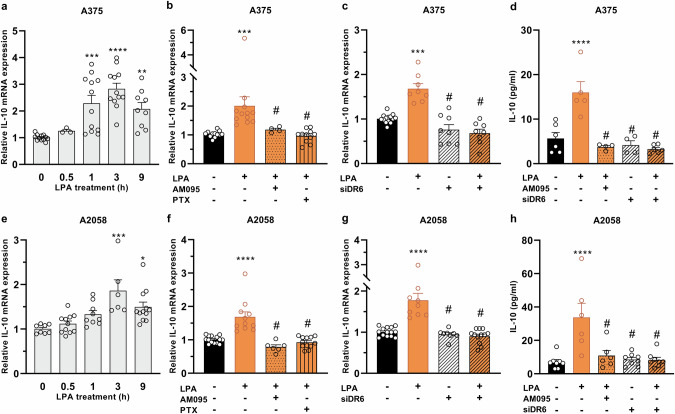
Regulation of LPA-induced IL-10 expression in human melanoma cells. Melanoma cells were treated with 10 µM LPA. Time-dependent induction of IL-10 expression was examined by qPCR (**a**, **e**). AM095 and PTX were used to examine the G_i_-coupled LPAR1 signaling (**b**, **f**), whereas DR6 was silenced by siRNA (**c**, **g**). siNC or siDR6-transfected A375 and A2058 melanoma cells were treated with 10 µM LPA or its vehicle for 12 h in the presence or absence of AM095. The level of IL-10 was determined in the cell supernatants (**d**, **h**). Statistical analysis was performed using one-way ANOVA and Dunnett’s posthoc test; *n* = 4–14, *P < 0.05, **P < 0.01, ***P < 0.001, ****P < 0.0001 vs control, ^#^*P* < 0.0008 vs. LPA alone.

LPA has been shown to modulate cytokine expression via activation of NF-κB [29]. Furthermore, NF-κB plays a central role in the regulation of cytokine networks in malignant cells [30] and upregulates interleukin 10 (IL-10) [31]. Since IL-10 plays a crucial role in the progression of melanoma, we investigated whether LPA could regulate IL-10 expression. Treatment of A2058 and A375 melanoma cells with 10 µM LPA increased IL-10 transcripts with a similar time course in both cell lines, resulting in maximal expression of IL-10 mRNA 3 h after LPA treatment (Fig. 3a, e). Our ability to inhibit LPA-induced upregulation of IL-10 with AM095 and PTX suggests its mediation via the LPAR1-G_i_ pathway (Fig. 3b, f).

Since our results suggest that LPA-induced IL-10 expression may be mediated by the same signaling steps that were previously identified in DR6 upregulation, we hypothesized that DR6 may be involved in the LPA-mediated production of IL-10. Thus, we examined whether silencing the expression of the DR6 gene using siRNA interfered with LPA-induced expression of IL-10 mRNA and found that blocking DR6 expression with siRNA reduced IL-10 mRNA levels. These results suggest that DR6 mediates LPA-induced IL-10 expression (Fig. 3c, g). We also showed that the LPA-induced secretion of IL-10 in melanoma cells is mediated by LPAR1 and DR6. Specifically, LPA treatment induced a 3- and 5-fold increase in IL-10 secretion in A375 (Fig. 3d) and A2058 cells (Fig. 3h), respectively. These effects were abolished completely by pharmacological inhibition of LPAR1 or siRNA-mediated silencing of DR6 expression (Fig. 3d, h), providing evidence for the involvement of the LPA-LPAR1-DR6 axis in increasing IL-10 secretion in melanoma.

### Correlation between LPAR1, DR6 and IL-10 expression in human melanoma

**Fig. 4:**
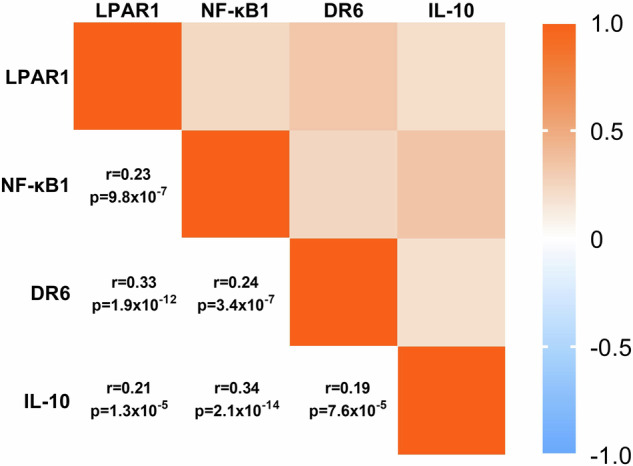
Correlation of LPA-DR6-IL-10 axis-related genes in patients with melanoma. Heatmap representing the correlation between the expression of LPAR1, NF-κB1, DR6, and IL-10 in melanoma tumor samples. *n* = 435. Statistical analysis was performed using Spearman rank correlation.

To verify the significance of the LPAR−NF-κB−DR6−IL-10 signaling cascade in human melanoma, we performed Spearman rank correlation analysis of melanoma patient data found in available databases. The correlations between the gene expression levels of LPAR1, NF-κB1, DR6, and IL-10 are presented in Fig. 4. Based on 435 melanoma samples, LPAR1 expression strongly correlated with the expression of NF-κB1 (Spearman’s *r* = 0.23, *P* = 9.8 × 10^−7^), DR6 (Spearman’s *r* = 0.33, *P* = 1.9 × 10^−12^) and IL-10 (Spearman’s *r* = 0.21, *P* = 1.3 × 10^−5^) (Fig. 4). Supporting our findings, expression of not only LPAR1 but also that of DR6 positively correlates with IL-10 (Spearman’s *r* = 0.19, *P* = 7.6 × 10^−5^) (Fig. 4). These results are consistent with our in vitro findings on the LPAR1−DR6−IL-10 signaling cascade in human melanoma.

### Effect of LPA on HLA-DR expression in melanoma

**Fig. 5:**
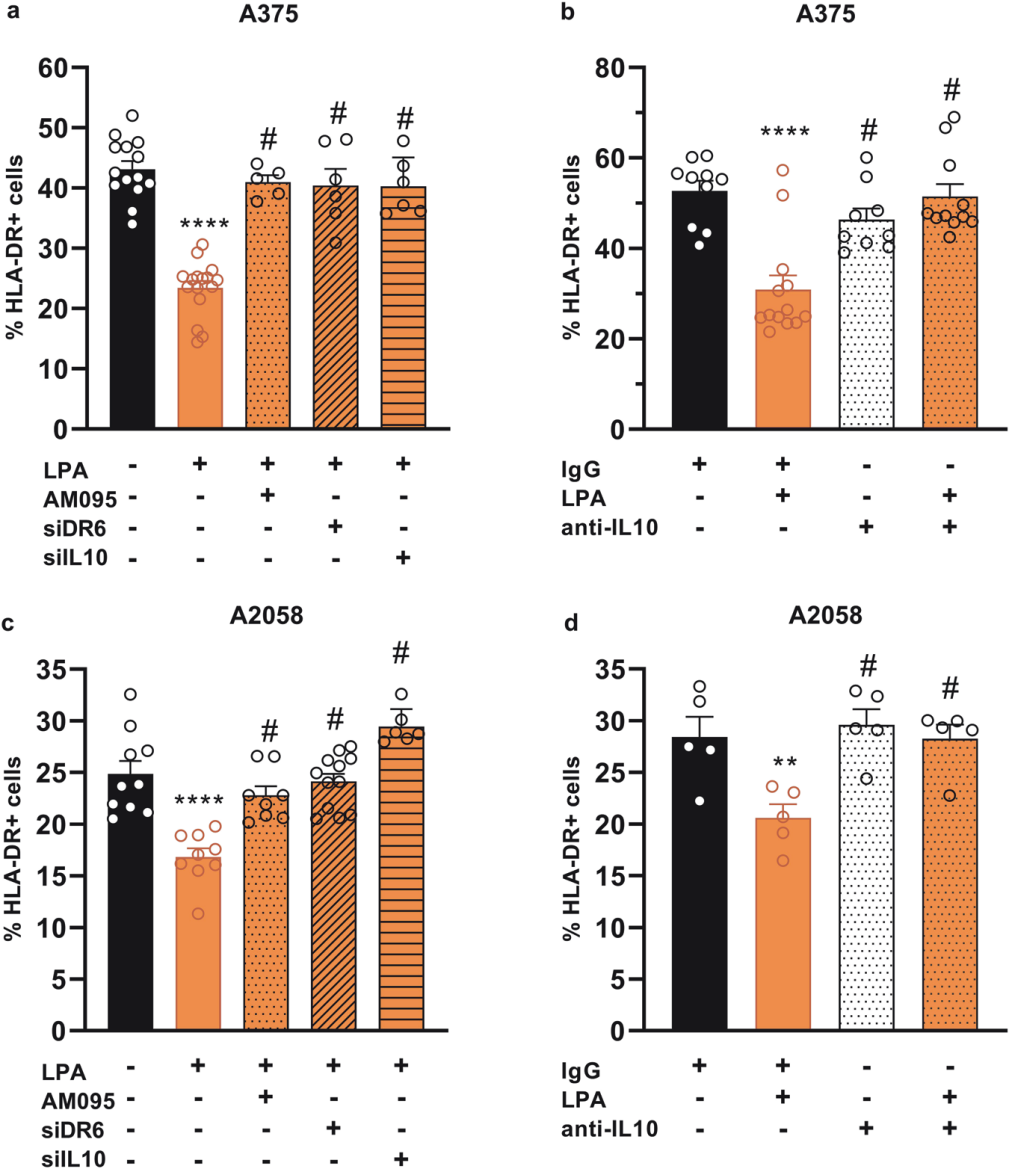
LPA-induced downregulation of HLA-DR in human melanoma. A375 and A2058 cells were treated with 10 µM LPA for 22 h and HLA-DR expression was measured by flow cytometry. The signaling pathway was examined using AM095, siDR6, or siIL10 (**a**, **c**). IL-10 was neutralized by an anti-IL10 monoclonal antibody and IgG1 kappa was used as an isotype control (**b**, **d**). Statistical analysis was performed using one-way ANOVA and Dunnett’s post hoc test; *n* = 5-14, **P < 0.01, ****P < 0.0001 vs. control, ^#^*P* < 0.05 vs. LPA.

**Fig. 6:**
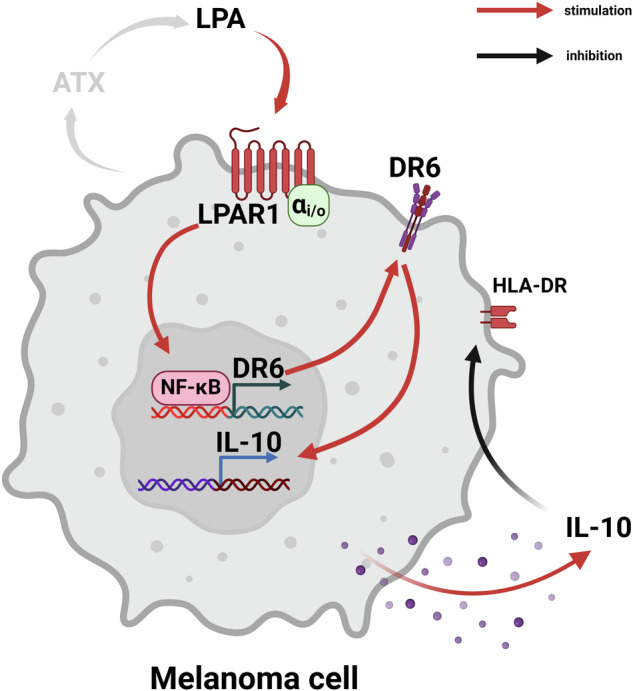
Signaling pathways of LPA-induced downregulation of HLA-DR in melanoma. LPA, via its G_i_-coupled LPAR1 receptor, activates NF-κB-mediated expression of DR6, which in turn induces the transcription and secretion of IL-10. LPA-mediated IL-10 release leads to the downregulation of HLA-DR antigen in human melanoma cells. Created with BioRender.com.

Next, we investigated whether the LPA-LPAR1-DR6 axis and subsequent IL-10 release affect the expression of HLA-DR in human melanoma. To do this, we treated A375 or A2058 cells with LPA in the absence and presence of the LPAR1 antagonist AM095, siRNA silencing of DR6 or IL-10, as well as in the presence of anti-IL-10 neutralizing monoclonal antibody or an IgG1 kappa isotype control. Treatment with LPA alone resulted in a marked downregulation of HLA-DR in both A375 (Fig. 5a) and A2058 melanoma cells (Fig. 5c), which was lost after pharmacological inhibition of LPAR1 by AM095 or siDR6-mediated silencing of DR6 expression. More importantly, silencing of IL-10 expression with siRNA (Supplementary Fig. S4) or blocking its effect with a neutralizing antibody totally abolished the effect of LPA on HLA-DR expression (Fig. 5). These results revealed that LPA downregulates HLA-DR expression in both melanoma cell lines by activating the LPAR1-DR6-IL-10 pathway (Fig. 6).

Finally, investigating the gene expression related to resistance to anti-PD-1 administration in melanoma tumors revealed that LPAR1 expression was significantly higher in anti-PD-1 non-responders than anti-PD-1 responders (AUC = 0.574, *P* = 1.4 × 10^−2^, strongest cutoff = 132), confirming the marked role of LPAR1 in the progression of melanoma. This analysis highlights the potential predictive value of LPAR1 expression in determining the response to anti-PD-1 therapy in melanoma patients. These findings suggest that increased expression levels of LPAR1 may be associated with worse therapeutic outcomes, underscoring the importance of further investigating this gene as a potential biomarker for immunotherapy response.

## Discussion

Melanoma is the most aggressive type of skin cancer, which can severely impact life expectancy when the disease has progressed to advanced stages [25]. While ICIs are a promising treatment for metastatic melanoma, ~50% of patients do not respond well to them [32]. In this study, we used human A375 and A2058 cell lines as representatives of primary and metastatic melanoma models, respectively, to better understand the molecular mechanism by which melanoma can escape the anti-tumor immune response.

Numerous experimental and clinical studies support the role of LPA in tumor development and progression. We hypothesized that LPA-induced IL-10 production contributes to altered expression of HLA antigens, thus modulating the anti-tumor immune response. Under healthy conditions, the concentration of LPA in the plasma is typically less than 100 nM, but in patients with certain types of malignancies, it is elevated several-fold [33]. Melanoma abundantly expresses autotaxin, the enzyme responsible for the biosynthesis of LPA [34]. Autotaxin creates an LPA-rich milieu in the melanoma microenvironment, which can influence tumor progression, metastasis formation, and anti-tumor immunity. The paracrine chemorepulsive effect of autotaxin-derived LPA on tumor-infiltrating lymphocytes and its role in the immune escape of melanoma has been well documented [35]. In the present study, we provide an alternative immune-escape mechanism involving an autocrine effect of LPA on the expression of HLA antigens in melanoma cells. Our results indicate that LPA downregulates HLA-DR, the expression of which strongly correlates with the therapeutic efficacy of ICIs [25]. This effect of LPA involves LPAR1-, G_i_-protein- and NF-κB-mediated upregulation of DR6 and consequent IL-10 release.

DR6 plays a multifaceted role in the progression of malignancies [12]. Although upregulated DR6 expression has been reported in several tumor types, including melanoma [10, 12], the exact role of DR6 in tumor biology remained obscure. Interestingly, a clinical study reported the association of DR6 upregulation with chemotherapy resistance in melanoma [36]. We show here that LPA regulates DR6 expression by acting on LPAR1. Furthermore, our results with PTX support the role of G_i_-protein in mediating the effect. The role of LPAR1 in LPA-induced cancer invasion and oncogenesis is well established [37]. In many primary tumors, elevated expression of LPAR1 correlates with increased proliferation and poor prognosis [37]. Moreover, LPAR1 is a crucial regulator of melanoma invasion, metastasis, and therapy resistance [38]. However, the present study is the first report on the role of LPA and LPAR1 in modulating HLA-DR expression and the effectiveness of anti-PD-1 therapy.

Our findings that LPA treatment increased DR6 promoter activity and mRNA expression after 30 min indicate that it acts as an immediate-early gene that responds to LPA. We examined the promoter sequence of DR6 and found that it contains conserved binding sites for NF-κB1 (p50). Our results showed that silencing NF-κB1 inhibited LPA-induced expression of DR6, confirming its role in mediating DR6 upregulation. NF-κB1 is an essential regulator of cell survival in several types of cancer including melanoma [39, 40]. Members of the NF-κB family, specifically p50 and p65, are overexpressed in melanoma cells compared to non-transformed melanocytes, supporting their contribution to cancer development [40]. Although our present study focused on DR6 and NF-κB in melanoma cells, their roles in immune cells are likely to be at least as important. For example, tumor derived DR6 modulates development of dendritic cells and thus activation of T cells [41]. NF-κB is likely to play an even more significant role in tumor progression as it appears to regulate the release of several chemokines and cytokines by melanoma cells [40]. Therefore, it is reasonable to hypothesize that in our experimental system NF-κB induces not only expression of DR6 but also that of IL-10. Unfortunately, due to methodological limitations, we were not able to address this question in the present study. Furthermore, given the central role of NF-κB in governing the immune response against tumor cells, NF-κB therefore represents an important therapeutic target for novel anti-cancer drugs [42, 43].

Although LPA is a known mediator of cytokine release [44], its role in regulating IL-10 expression by tumor cells has not been described previously. In LPS-stimulated human dendritic cells and macrophages, LPA increases IL-10 release with subsequent inhibition of TNF-α production [45]. IL-10 is an important immunosuppressive cytokine that is frequently upregulated in the tumor microenvironment [46, 47]. In clinical studies, IL-10 expression and production correlate with melanoma progression [23]. Sato et al. reported that transformed melanocytes are a major source of IL-10 production in melanoma metastases [47]. Moreover, DR6 also serves as an immunosuppressive factor involved in inhibiting the proliferation and migration of B and T cells, supporting its role in the induction of tumor survival and progression [12]. Our study is the first to report LPA-induced IL-10 production by tumor cells and identifies LPAR1-dependent upregulation of DR6 receptors in mediating this effect.

IL-10 can inhibit essential steps in immune detection by decreasing the cell surface expression of HLA class I and II antigens and the intercellular adhesion molecule-1 (ICAM-1) in melanoma cells [48]. The efficacy of immunotherapy depends on the identification of the cell surface antigens by T cells [25]. In cancerous diseases, low expression of the MHC-II isotype antigen HLA-DR is often associated with poor survival [25]. Although HLA-DR expression alone does not appear to determine the prognosis, it strongly influences the therapeutic efficacy of ICIs for the treatment of melanoma [25]. Recently, numerous retrospective clinical studies proved that tumor cell HLA-DR positivity is associated with a higher response rate for ICI immunotherapy in patients with advanced melanoma [25]. In response to ICI, significantly better progression-free and overall survival were observed in the HLA-DR positive tumors compared to tumors that were HLA-DR negative (tumors with less than 5% HLA-DR^+^ melanoma cells) [25]. Our results established that LPA significantly downregulates the expression of HLA-DR in human melanoma cells, which is a novel mechanism of LPA-induced immune escape. This effect is mediated predominantly by upregulation of DR6 and consequent release of IL-10, although a direct effect of DR6 on HLA-DR cannot be excluded based on our experimental data. Furthermore, we found that LPAR1 expression negatively correlates with the effectiveness of anti-PD-1 therapy, suggesting a role of the LPA-induced downregulation of HLA-DR in therapy resistance. Interestingly, Kovács et al. reported recently significantly higher expression of LPAR1 in non-responders to ICI therapy in a cohort of different tumor types [28]. Additionally, Konen et al. demonstrated that ATX and LPA are upregulated in anti-PD-1 resistant non-small cell lung cancer and negatively correlated with the number of infiltrating CD8^+^ T cells [49], indicating that increased LPA levels negatively affect the response to ICI. The present study reveals a connection between the elevated LPA concentration and IL-10 production, which in turn diminishes the HLA-DR expression in melanoma. Furthermore, our results demonstrated that the siRNA-mediated knockdown of DR6 abolished the effect of LPA on IL-10 expression and release, reinforcing the central role of DR6 in LPA-induced cytokine release by tumor cells.

In summary, the present study demonstrates that LPA increases DR6 expression in a G_i_-coupled LPAR1- and NF-κB-dependent manner. Moreover, LPA is a potent regulator of IL-10 gene transcription and protein release via DR6, resulting in decreased HLA-DR expression in melanoma cells (Fig. 6). Because IL-10 plays an important role in the immune escape of tumors and neutralizing IL-10 has been proposed as a novel anti-tumor therapy [50], downregulation of HLA-DR through LPA-induced IL-10 production might be an important pathway in the progression, metastasis, and immune escape of melanoma.

## Supplementary information

Supplementary Figure S1

Supplementary Figure S2

Supplementary Figure S3

Supplementary Figure S4

Supporting information
