# Supplementary figures and images for: LPA suppresses HLA-DR expression in human melanoma cells: a potential immune escape mechanism involving LPAR1 and DR6-mediated release of IL-10
Source: Acta Pharmacol Sin. 2024 Aug 26;46(1):222–30. doi: 10.1038/s41401-024-01373-x (PMC11696067; doi:10.1038/s41401-024-01373-x)

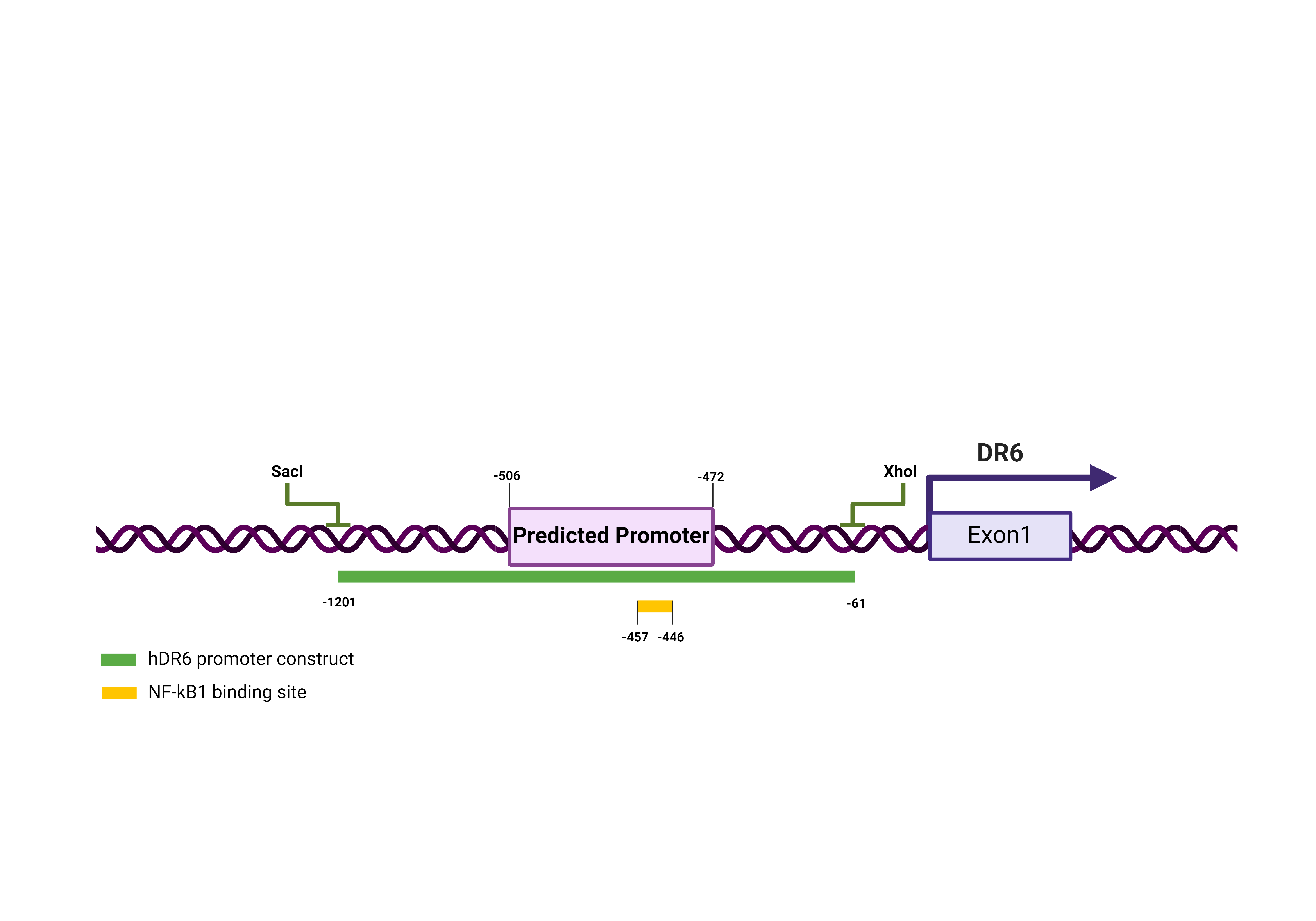

Supplement: Supplementary file 1 — Supplementary Figure S1 [file 41401_2024_1373_MOESM1_ESM.jpeg]

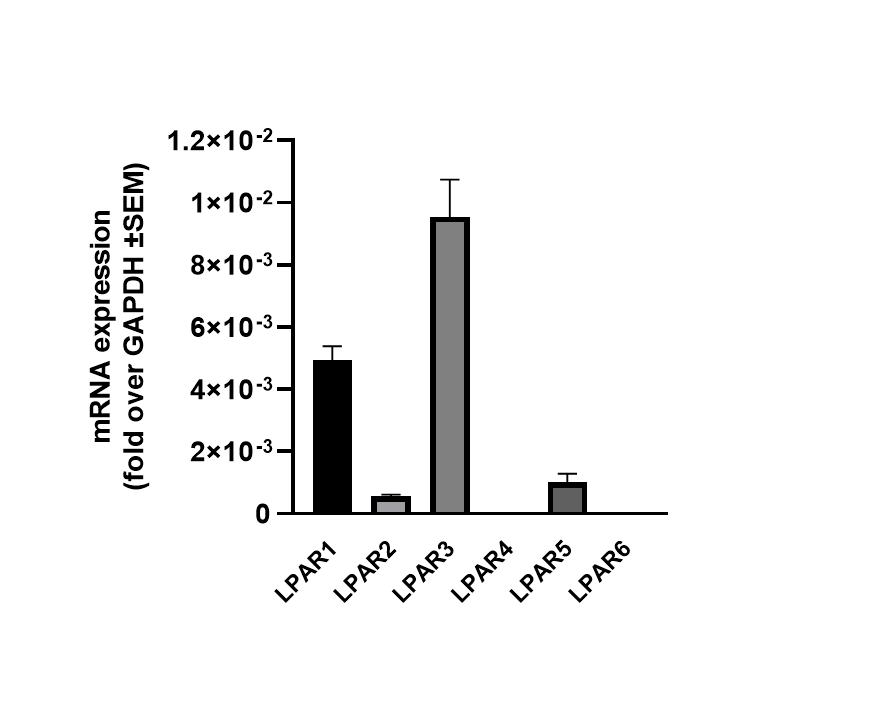

Supplement: Supplementary file 2 — Supplementary Figure S2 [file 41401_2024_1373_MOESM2_ESM.tif]

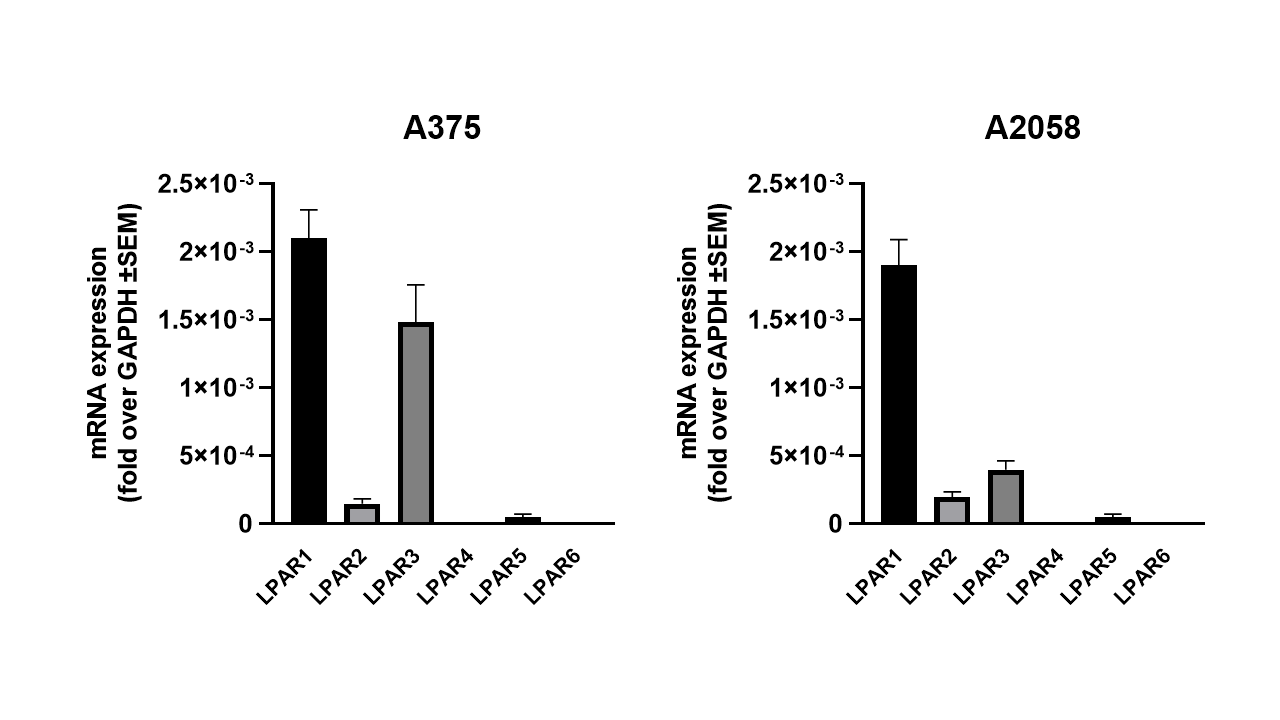

Supplement: Supplementary file 3 — Supplementary Figure S3 [file 41401_2024_1373_MOESM3_ESM.tif]

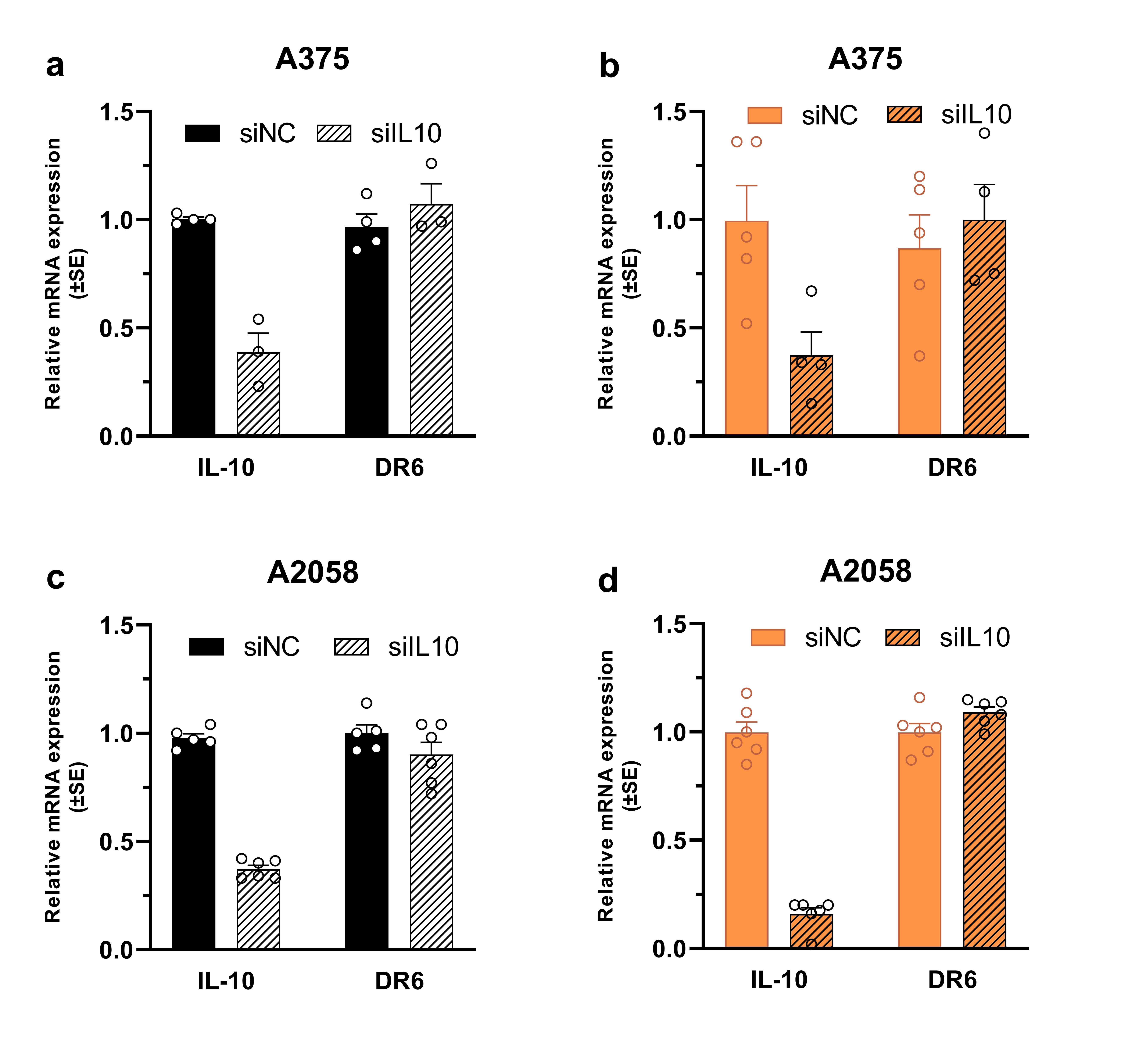

Supplement: Supplementary file 4 — Supplementary Figure S4 [file 41401_2024_1373_MOESM4_ESM.jpg]
