# Supplementary material for: LPA suppresses HLA-DR expression in human melanoma cells: a potential immune escape mechanism involving LPAR1 and DR6-mediated release of IL-10
Source: Acta Pharmacol Sin. 2024 Aug 26;46(1):222–30. doi: 10.1038/s41401-024-01373-x (PMC11696067; doi:10.1038/s41401-024-01373-x)
Supplement: Supplementary file 5 — Supporting information [file 41401_2024_1373_MOESM5_ESM.docx]

**Supporting information**

**Supplementary Figure S1**. The putative promoter region of human DR6 was identified using the Berkley NNPP program. Created with [BioRender.com](https://biorender.com/).

**Supplementary Figure S2.** LPAR mRNA profile of HEK293T cells was quantified by qPCR. *n*=6

**Supplementary Figure S3.** LPAR mRNA profile of A375 and A2058 cells was quantified by qPCR. *n*=6

**Supplementary Figure S4.** IL-10 and DR6 mRNA expression of siNC or siIL10 transfected melanoma cells measured by qPCR, in the absence (a, c) or the presence (b, d) of 10 µM LPA. *n*=3-6
